# Nationwide study on clinical impact of early tumor necrosis factor-α inhibitors following first intestinal resection in biologics-naïve Crohn’s disease patients

**DOI:** 10.1038/s41598-025-88364-y

**Published:** 2025-04-11

**Authors:** Ji Eun Na, Sung Hoon Jung, Arum Choi, Sukil Kim, Tae-Oh Kim

**Affiliations:** 1 Division of Gastroenterology, Department of Internal Medicine, Haeundae Paik Hospital, Inje University College of Medicine, 875 Haeundae-ro, Haeundae-gu, Busan, 48108 Korea; 2 Division of Gastroenterology, Department of Internal Medicine, Eunpyeong St. Mary’s Hospital, College of Medicine, The Catholic University of Korea, 1021, Tongil-ro, Eunpyeong-gu, Seoul, 03312 Republic of Korea; 3 Department of Preventive Medicine and Public Health, College of Medicine, The Catholic University of Korea, Seoul, Korea

**Keywords:** Crohn’s disease, Postoperative management, Early versus late anti-tumor necrosis factor therapy, Gastroenterology, Gastrointestinal diseases

## Abstract

In Crohn’s disease (CD) patients who have undergone surgery, postoperative recurrence remains a challenge, and there is a lack of investigation into the impact of early anti-tumor necrosis factor (TNF) therapy following surgery on clinical outcomes compared to late use of anti-TNF agents. Utilizing the Health Insurance Review and Assessment database, we conducted a retrospective cohort study comprising 481 CD patients who underwent their first intestinal resection and were naïve to preoperative biological therapy from 2010 to 2018. Patients initiating anti-TNF agents early (within one year post-surgery) were compared to those starting later for prognosis. Clinical outcomes, including biologics switching and surgical recurrence, were assessed. The late start group (n = 290) exhibited a higher surgical recurrence rate (27.9% vs. 18.3%, *p*-value = 0.021), while biologics switching rates did not significantly differ (8.3% vs. 12.6%, *p*-value = 0.167) compared to the early start group (n = 191). Kaplan–Meier curves revealed no significant differences in the risk of biologics switching (*p*-value = 0.319) or surgical recurrence (*p*-value = 0.380) between the early and late start groups. This study investigated the potential role of early anti-TNF therapy after first intestinal resection in biologics-naïve CD patients compared to late initiation. Further refined prospective research is warranted to validate these comparisons.

## Introduction

The advancement of medical treatment using biological agents has improved the long-term prognosis of Crohn’s disease (CD) patients^1^. Despite this progress, approximately 30% of patients with CD still undergo surgery due to complications such as strictures, abscesses, or fistulas, as well as treatment failures^2,3^. Furthermore, since surgery does not equate to a cure, it is reported that a majority of CD patients experience endoscopic recurrence within the third year after surgery^4^. Within five years post-surgery, 25% of patients undergo additional surgery^5^. To mitigate the risk of postoperative recurrence, current guidelines recommend postoperative prevention therapy, which includes metronidazole, immunomodulators, and anti-tumor necrosis factors (TNF) agents according to the stratified risk. Patients are categorized into high-risk or low-risk based on factors such as young age, current smoking, a history of at least two previous surgeries, penetrating behavior, or a history of perianal disease^6–10^. Furthermore, even without these risk factors, postoperative intervention using immunomodulators or anti-TNF agents is recommended if endoscopic recurrence with Rutgeerts score of i2a or higher is confirmed within 6–12 months after surgery^6–10^.

Recent data indicates that initiating biological therapy early after surgery, compared to those not receiving biological therapy, may decrease the risk of endoscopic recurrence and enhance long-term clinical outcomes, such as reducing hospitalization and surgery rates^11,12^. Furthermore, for CD patients with previous exposure to biologics, the early or continued use of biological agents post-surgery has been suggested as a preventive measure against endoscopic recurrence and a means to decrease the risk of clinical recurrence^13,14^.

However, there have been no reports on the impact of the early start of biological therapy after the first bowel resection in biologics-naïve CD patients, compared to the late initiation of biological therapy, concerning its influence on clinical outcomes. Therefore, we aimed to investigate how the early use of biological agents after the first bowel resection in bio-naïve CD patients affects clinical outcomes compared to the later use of biological agents, based on national data.

## Methods

### Data source

Data were sourced from a population-based retrospective cohort using the Health Insurance Review and Assessment (HIRA) database. CD patients were identified using diagnostic codes from the International Classification of Diseases, 10th revision (ICD-10), and the V code in the rare intractable diseases (RID) database. The study received approval from the Institutional Review Board of Haeundae Paik Hospital, with the IRB approval number 2023-07-032. All protocols followed relevant guidelines, and the requirement for the acquisition of informed consent was waived by the Institutional Review Board of Haeundae Paik Hospital due to the study’s retrospective nature, as only de-identified data were collected.

### Study population

The study population consisted of CD patients aged 18 and above, identified using the ICD-10 codes K50 and V code 130 from 2010 to 2018. Patients who underwent first intestinal resection were screened with exclusions applied for the following cases: (1) patients with a history of biological therapy before first intestinal resection, (2) patients who did not receive post-surgery biological therapy, (3) patients who initiated biological therapy after surgical recurrence (second intestinal resection), and (4) patients who received biological therapy for less than six months. Biologics therapy is generally indicated for patients with moderate to severe active CD (Crohn’s Disease Activity Index ≥ 220) who have failed to respond to at least two conventional therapies, including steroids or immunomodulators, or for whom such treatments are intolerable or contraindicated. Based on the initiation of biological therapy within one year of surgery, we categorized patients into the early and late start groups. Intestinal resection was identified through specific surgical codes, including small bowel resection and anastomosis (Q2650 and Q2651) and colectomy [QA(or 2)67(1 ~ 3 or 9), Q1261, Q1262, Q2691, Q2761, Q2771, QA(or 2)92(1 ~ 6 or 8)]. During the study period, the first-line biologics available were only anti-TNF agents, specifically infliximab and adalimumab.

### Outcomes

The primary outcome compared clinical outcomes, specifically the occurrence of biologics switching and surgical recurrence during the follow-up period between the early and late start groups. The secondary outcome involved analyzing factors associated with biologics switching and surgical recurrence. Biologics switching was defined as initiating infliximab or adalimumab but subsequently changing to another biologics. Surgical recurrence was defined as additional intestinal resection after the first intestinal resection. The index date was based on the surgery date, and the follow-up was extended until the end of the study period in December 2018. Clinical outcomes were censored for events occurring after the initiation of biologics.

### Covariates

The following variables were considered as potential confounders and were collected: gender, age at intestinal resection, age at biological therapy, history of perianal surgery (Q2881 ~ 3, Q2950, Q2974 ~ 9), anti-TNF agents type after intestinal resection, concomitant medication [5-aminosalicylic acid (5-ASA), immunomodulators with methotrexate or thiopurines, steroid, and anti-TNF agents], and the hospital scale (tertiary or general/community hospitals).

### Statistical analysis

The continuous variables were analyzed using Student’s t-test, and categorical variables were assessed using the chi-squared test. The occurrence rates of biologics switching or surgical recurrence between the early and late start groups of anti-TNF agents during the follow-up period were compared using the chi-squared test. Cumulative occurrences of events were compared using Kaplan–Meier curves with log-rank *p*-value tests. Cox proportional hazards regression analysis was employed to identify factors associated with biologics switching or surgical recurrence. Only factors with a *p*-value less than 0.1 in the univariable analysis, including the interesting variable of early or late start of anti-TNF agents, were included in the multivariable analysis. Statistical significance was set at a *p*-value less than 0.05. The statistical analysis used R version 4.0.3 (R Foundation for Statistical Computing, Vienna, Austria) and SAS version 7.1 (SAS Institute Inc., Cary, NC).

## Results

### Study population

**Fig. 1:**
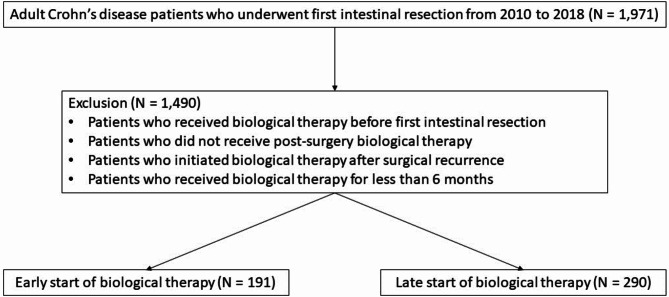
Flowchart for patient selection.

From 2010 to 2018, 1,971 adult CD patients who underwent first initial intestinal resection were identified. After applying the exclusion criteria and excluding 1,490 patients, 481 adult CD patients who were biologics-naïve and had experienced their first intestinal resection were included in the study. Among them, 191 were classified into the early start group, while 290 were classified into the late start group (Fig. 1).

When comparing the baseline characteristics between the early and late start groups, no significant differences were observed in gender and age at the time of intestinal resection (Table 1). However, the age at biological therapy was significantly higher in the late start group, with a mean of 33.5 years, compared to 30.7 years in the early start group. The early start group initiated biological therapy at a mean of 4.8 months after surgery, while the late start group started it at a mean of 39.6 months after surgery. There were no significant differences between the two groups in a history of perianal surgery and concomitant medication. The follow-up duration, measured from the index date (the surgery date), was longer in the late start group, with a mean of 72.2 months compared to 44.1 months in the early start group.

**Table 1:** Baseline characteristics of the study population.

Variable	Early start (N = 191)	Late start (N = 290)	*P*-value
Gender			0.956
Male	141 (73.8)	216 (74.5)	
Female	50 (26.2)	74 (25.5)	
Age at intestinal resection, years	30.4 ± 12.2	30.3 ± 11.8	0.952
Age at biological therapy, years	30.7 ± 12.1	33.5 ± 12.0	0.015
Duration between intestinal resection and biological therapy, months	4.8 ± 3.8	39.6 ± 23.0	< 0.001
History of perianal surgery	9 (4.7)	28 (9.7)	0.069
Biological therapy after intestinal resection			0.727
Infliximab	53 (27.8)	86 (29.7)	
Adalimumab	138 (72.2)	204 (70.3)	
Concomitant medication			
5-ASA	182 (95.3)	279 (96.2)	0.794
Immunomodulators	184 (96.3)	283 (97.6)	0.602
Steroid	183 (95.8)	277 (95.5)	0.128
Hospital scale			< 0.001
Tertiary hospitals	126 (66.0)	231 (79.7)	
General/community hospitals	65 (34.0)	59 (20.3)	
Follow-up duration, months	44.1 ± 26.9	72.2 ± 27.0	< 0.001

Variables are expressed as mean ± standard deviation or number (%).

5-ASA, 5-aminosalicylic acid.

### Comparison of clinical outcomes

**Fig. 2:**
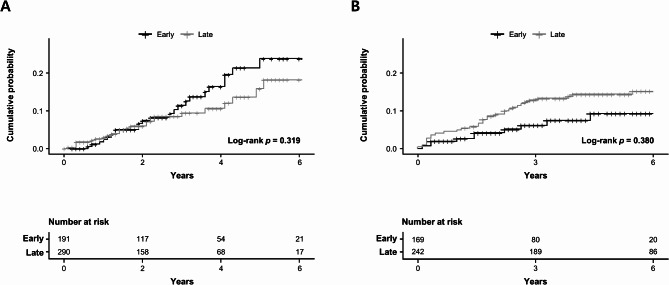
Kaplan–Meier curves illustrate clinical outcomes in Crohn’s disease patients with the early start of biologics compared to a late start. (**A**) Biologics switching; (**B**) Surgical recurrence.

During the follow-up period, biologics switching occurred in 24 (12.6%) of 191 patients in the early start group and 24 (8.3%) of 290 patients in the late start group, with no significant difference between the two groups (*p*-value = 0.167) (Table 2). The cumulative incidence also did not show a significant difference (*p*-value = 0.319) (Fig. 2).

**Table 2:** Clinical outcomes based on early versus late start of anti-tumor necrosis factor therapy.

	Early start (N = 191)	Late start (N = 290)	*P*-value
Biologics switching	24 (12.6)	24 (8.3)	0.167
Surgical recurrence	35 (18.3)	81 (27.9)	0.021

Variables are expressed as numbers (%).

Surgical recurrence was observed in 35 (18.3%) in the early start group and 81 (27.9%) in the late start group, demonstrating a significantly higher occurrence rate in the late start group (*p*-value = 0.021). However, the cumulative incidence between the two groups did not reach a statistically significant difference (*p*-value = 0.380).

### Risk factors for clinical outcomes

The risk of biologics switching [Hazard Ratio, HR: 0.68, 95% Confidence Interval, CI: 0.39–1.21] and surgical recurrence (HR 1.77, 95% CI 0.79–3.98) did not exhibit significant differences between the group that initiated anti-TNF agents early and the group that started them late (Table 3). The risk of biologics switching was higher in tertiary hospitals compared to general or community hospitals (HR 0.31, 95% CI 0.13–0.73). In univariable analysis, the risk of surgical recurrence was higher with younger age at intestinal resection (HR 0.97, 95% CI 0.94–0.99); this significance did not persist in multivariable analysis.

**Table 3:** Risk factors associated with clinical outcomes in Crohn’s disease patients with early and late start of anti-tumor necrosis factor therapy.

	Biologics switching						Surgical recurrence					
	Univariable			Multivariable			Univariable			Multivariable		
	HR	95% CI	*P*-value	HR	95% CI	*P*-value	HR	95% CI	*P*-value	HR	95% CI	*P*-value
Early*/Late start	0.75	0.43–1.32	0.319	0.68	0.39–1.21	0.192	1.34	0.70–2.56	0.380	1.77	0.79–3.98	0.168
Male*/Female	1.44	0.78–2.66	0.240				1.13	0.59–2.15	0.709			
Age at intestinal resection	1.00	0.98–1.03	0.793				0.97	0.94–0.99	0.036	1.08	0.87–1.35	0.486
Age at biological therapy	1.00	0.98–1.03	0.904				0.97	0.94–0.99	0.036	0.91	0.74–1.11	0.342
History of perianal surgery	0.62	0.15–2.56	0.508				1.29	0.51–3.26	0.592			
IFX*/ADA	1.06	0.55–2.05	0.857				0.67	0.33–1.35	0.263			
5-ASA No*/Yes	NA	NA	NA				NA	NA	NA			
IMs No*/Yes	1.88	0.26–13.64	0.533				1.31	0.18–9.51	0.790			
Steroid No*/Yes	NA	NA	NA				2.25	0.31–16.29	0.424			
Tertiary*/General or community	0.32	0.14–0.76	< 0.010	0.31	0.13–0.73	< 0.010	0.83	0.42–1.64	0.599			

IFX, infliximab; ADA, adalimumab; 5-ASA, 5-aminosalicylic acid; IMs, immunomodulators; NA, not available.

*Reference.

## Discussion

In biologics-naïve adult CD patients who underwent their first intestinal resection, initiating anti-TNF agents within one year after surgery was associated with a lower surgical recurrence rate than starting them later (18.3% versus 27.9%). While the occurrence rate of biologics switching was higher in the early start group compared to the late start group, this difference did not show statistical significance (12.6% versus 8.3%).

This paper is the first study to compare the clinical outcomes of early biologics within one year after the first intestinal resection with the late biologics start group in adult biologics-naïve CD patients. Moreover, the use of nationwide data aimed to minimize selection bias. The role of early biologics in CD patients after surgery has been unclear until now. Two retrospective studies have addressed this issue. In one study, the group that received early biologics within one year after surgery had a lower risk of surgery or hospitalization than the group that did not receive biological therapy^12^. Another study found that anti-TNF therapy within four weeks after surgery was only associated with a lower risk of postoperative recurrence, and other classes of biologics or biologics within 12 or 24 weeks did not prove to reduce the risk of postoperative recurrence compared to the group not receiving biologics^11^. However, it is crucial to note that the evaluation timing and method for postoperative recurrence after biologics administration varied in the latter study, requiring caution in interpretation. Furthermore, in a prospective study, there was no difference in endoscopic remission or recurrence at 18 months between patients at high risk for recurrence who initiated immediate adalimumab and those who started only thiopurine first, followed by a step-up to adalimumab combination therapy based on a colonoscopy at six months with Rutgeerts score ≥ i2^15^. In summary, previous studies observed that early biologics may improve long-term outcomes compared to the group not receiving biologics, and immediate anti-TNF agents after surgery had little impact on endoscopic recurrence compared to step-up based on a colonoscopy at six months. This paper provides data comparing the value of early and late biologics in biologics-naïve CD patients after surgery, offering additional insights for future research in this area.

While the definition of early biologics in patients who have not undergone surgery varies from 1 to 3 years in previous literature, numerous studies have already demonstrated that early biologics improve clinical outcomes such as clinical remission, steroid-free remission, mucosal healing, relapse, complications, or the need for surgery^16–25^. Moreover, recent evidence suggests that early biologics may result in overall cost savings in healthcare expenditures^26,27^. In this paper, the group starting early biologics exhibited a lower incidence rate of surgical recurrence than the late start group. However, statistical significance was not demonstrated in cumulative risk over time. Perhaps the lack of differentiation between high-risk (such as young age, current smoking, a history of at least two previous surgeries, penetrating behavior, or a history of perianal disease) and low-risk factors for postoperative recurrence, the absence of information on postoperative colonoscopy, and difficulty assessing the remaining inflammation or disease activity after surgery may affect the limitation for comparison. Despite these limitations, our results suggest a potentially beneficial role of early biologics. Further research is warranted to explore this possibility more comprehensively.

In this study, the higher risk of biologics switching in tertiary hospitals compared to general/community hospitals is thought to be influenced by the severity among the hospitals. In univariate analysis, the older age at the time of intestinal resection was associated with a lower risk of surgical recurrence, consistent with the previous report^8^.

This study has several limitations. The absence of information on key factors related to postoperative prognosis and the rationale for step-up therapy introduces the potential for selection bias. However, efforts were made to minimize selection bias by utilizing national data, and we aimed to provide a comparative analysis of the role of early biologics against late biologics for the first time. Although the study compared early and late initiation of biologic therapy after the first intestinal resection in biologics-naïve CD patients, the surgery rate has decreased with the biologics era^3,28^. Considering that surgery is not a curative treatment in CD, and the current trends favor proactive intervention based on the treat-to-target strategy for avoiding surgery^29^, future research may be needed to explore the role of postoperative biologics in patients with CD who have previous biologics experience^14^.

In conclusion, this paper observed that early use of anti-TNF agents after the first intestinal resection in biologics-naïve CD patients was associated with a lower surgical recurrence rate than late use, although statistical power was limited. Based on this study, more refined prospective research is needed.

## Data Availability

Data is available on request. The data underlying this article will be shared upon reasonable request to the corresponding author.
